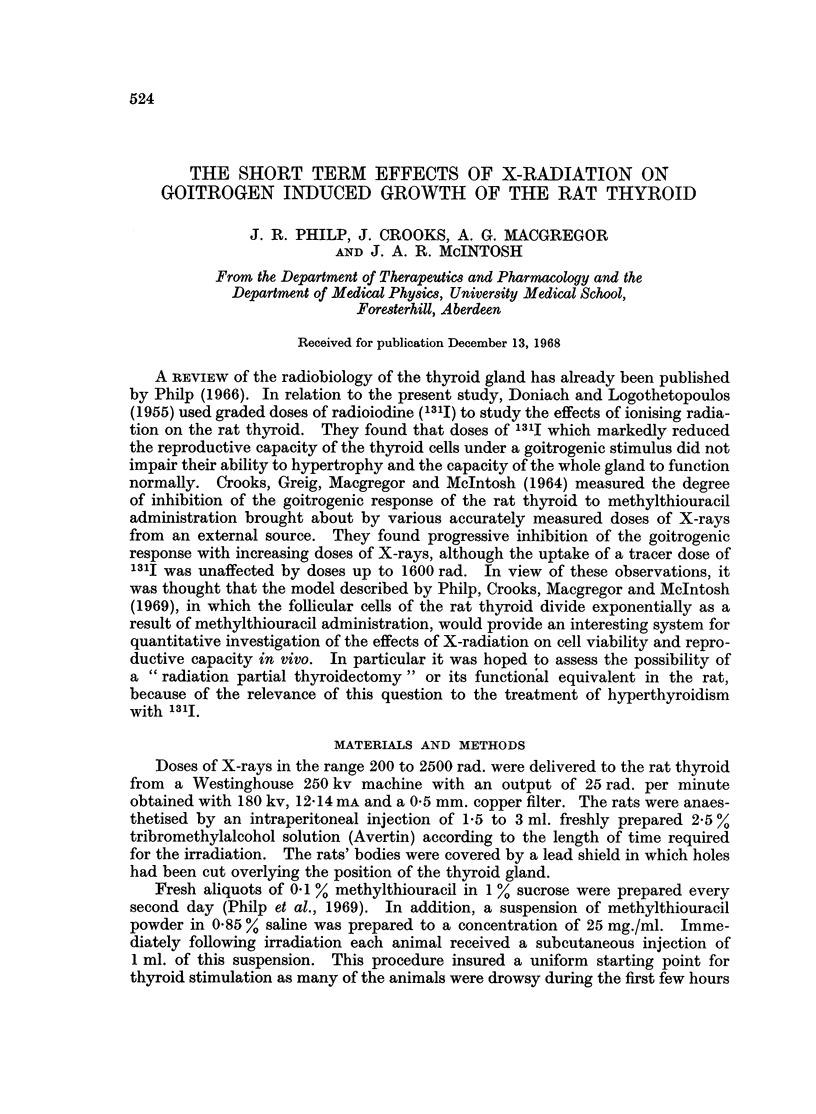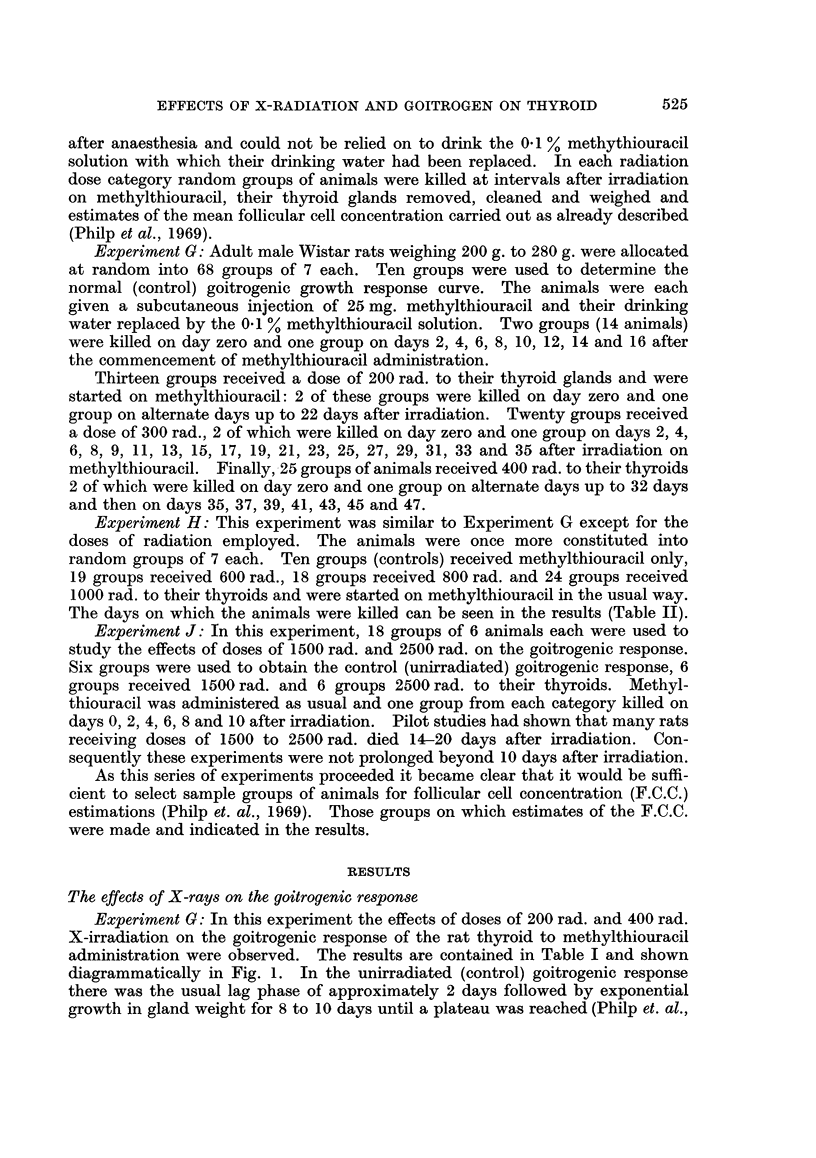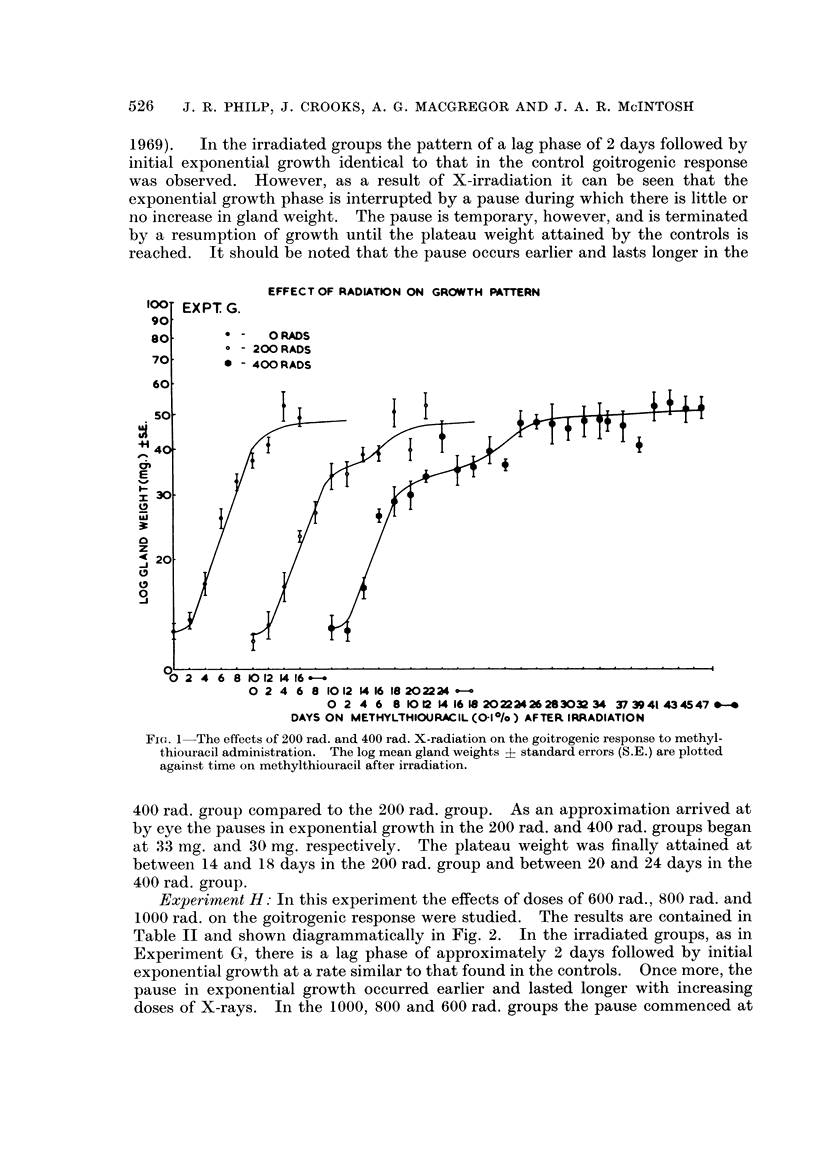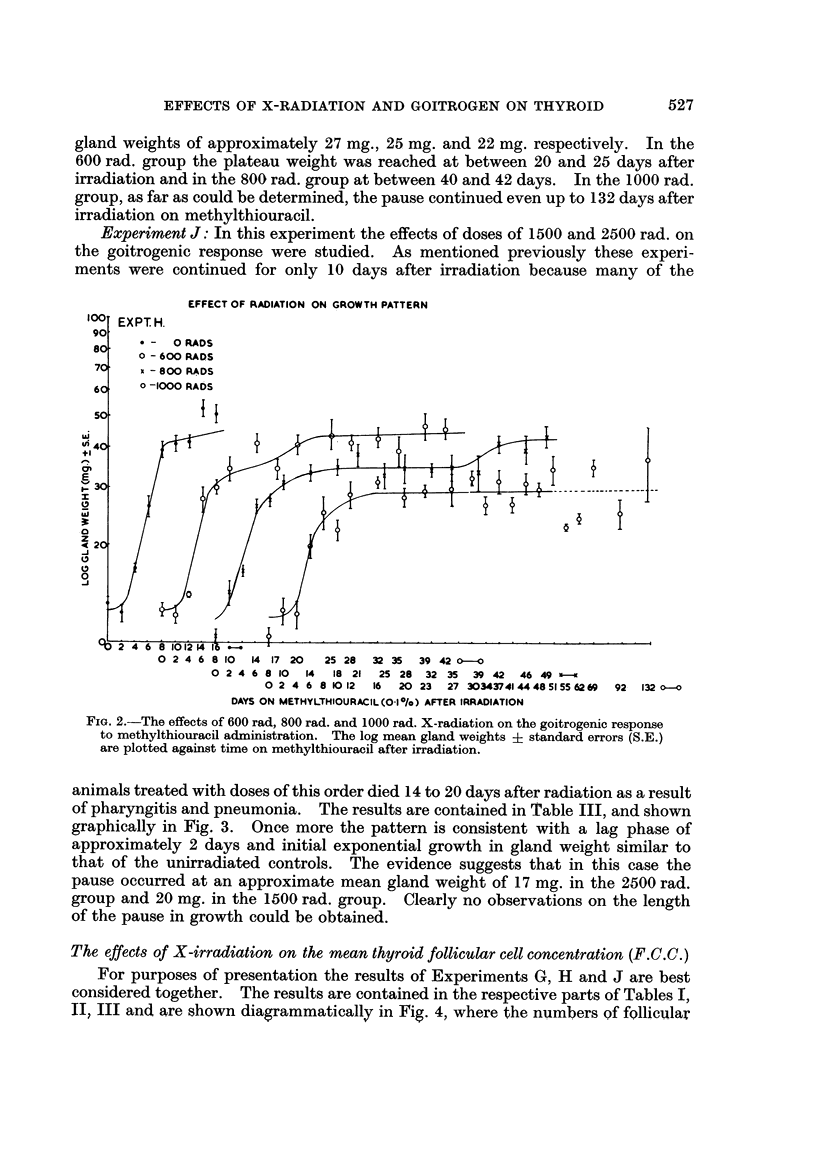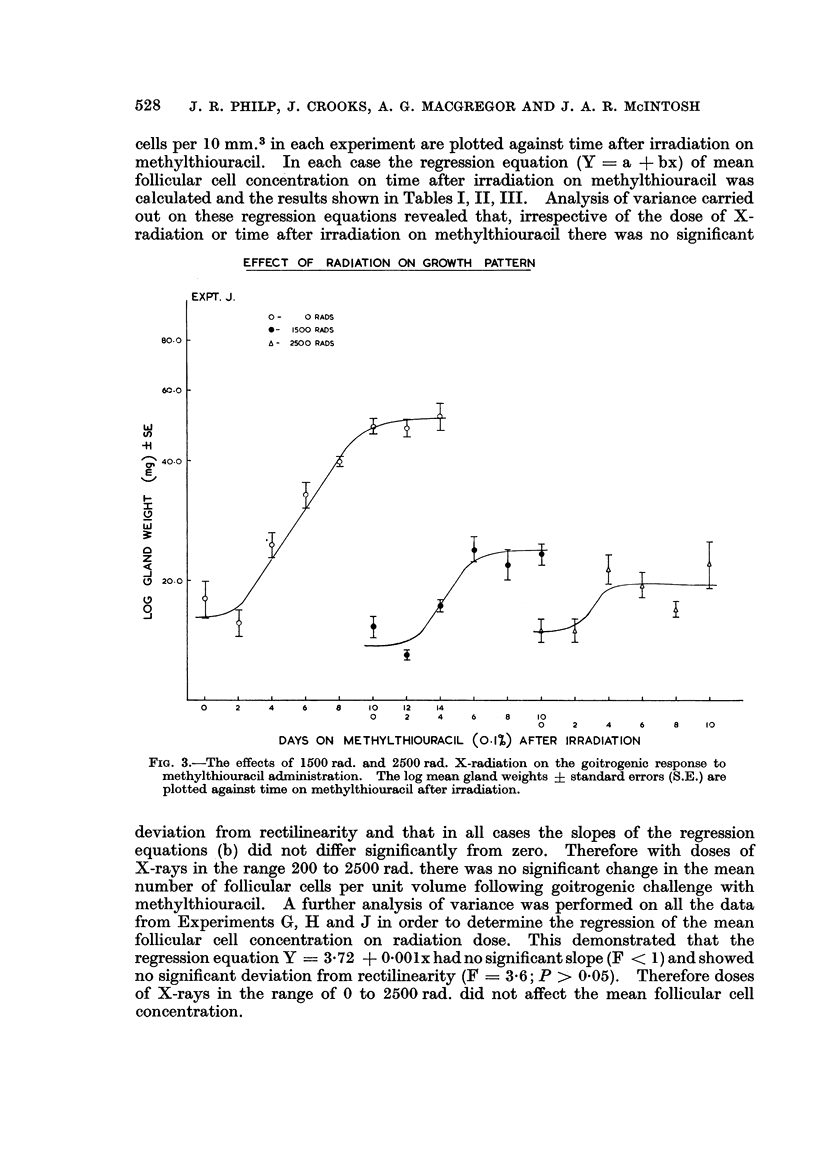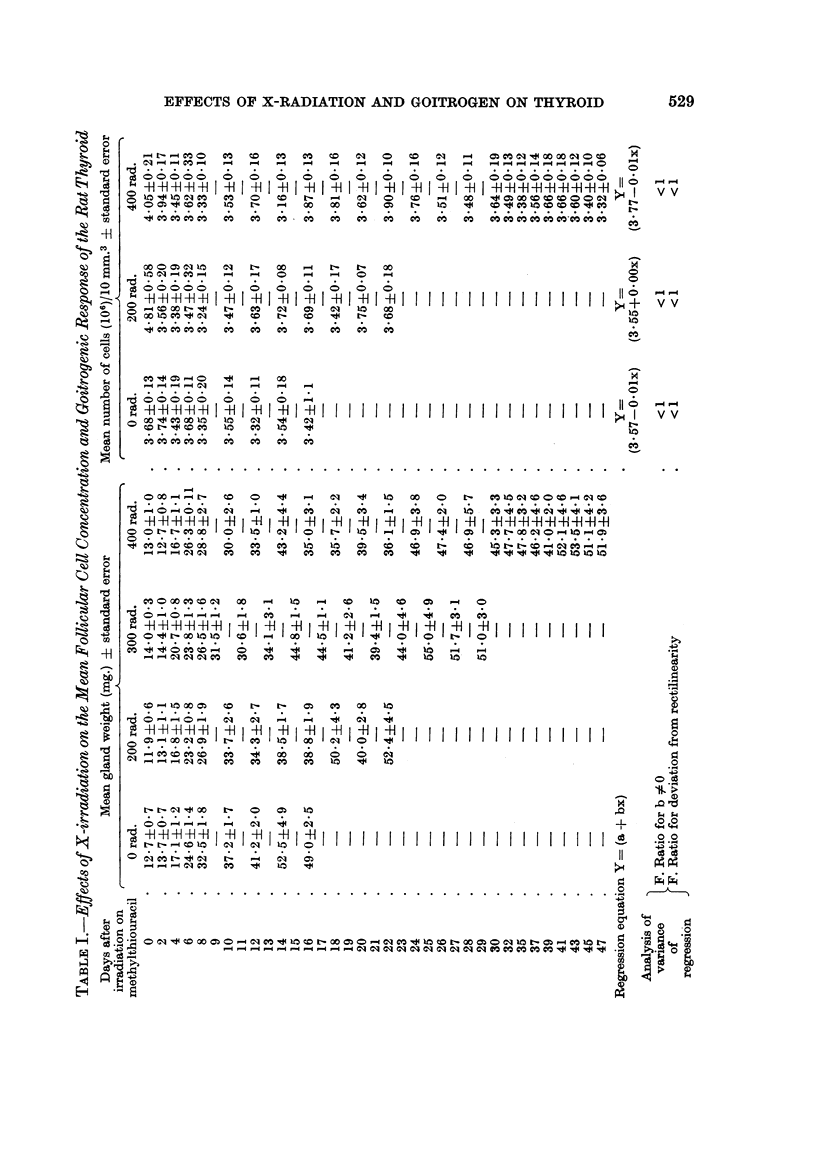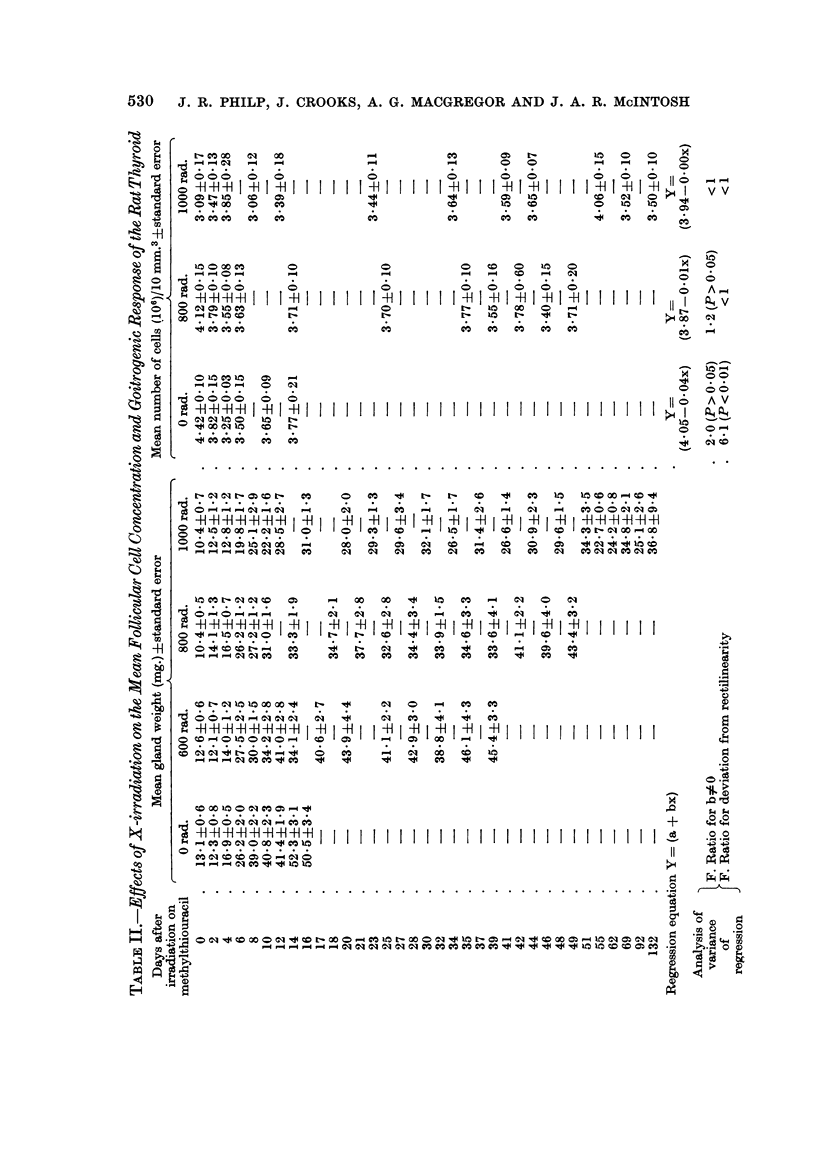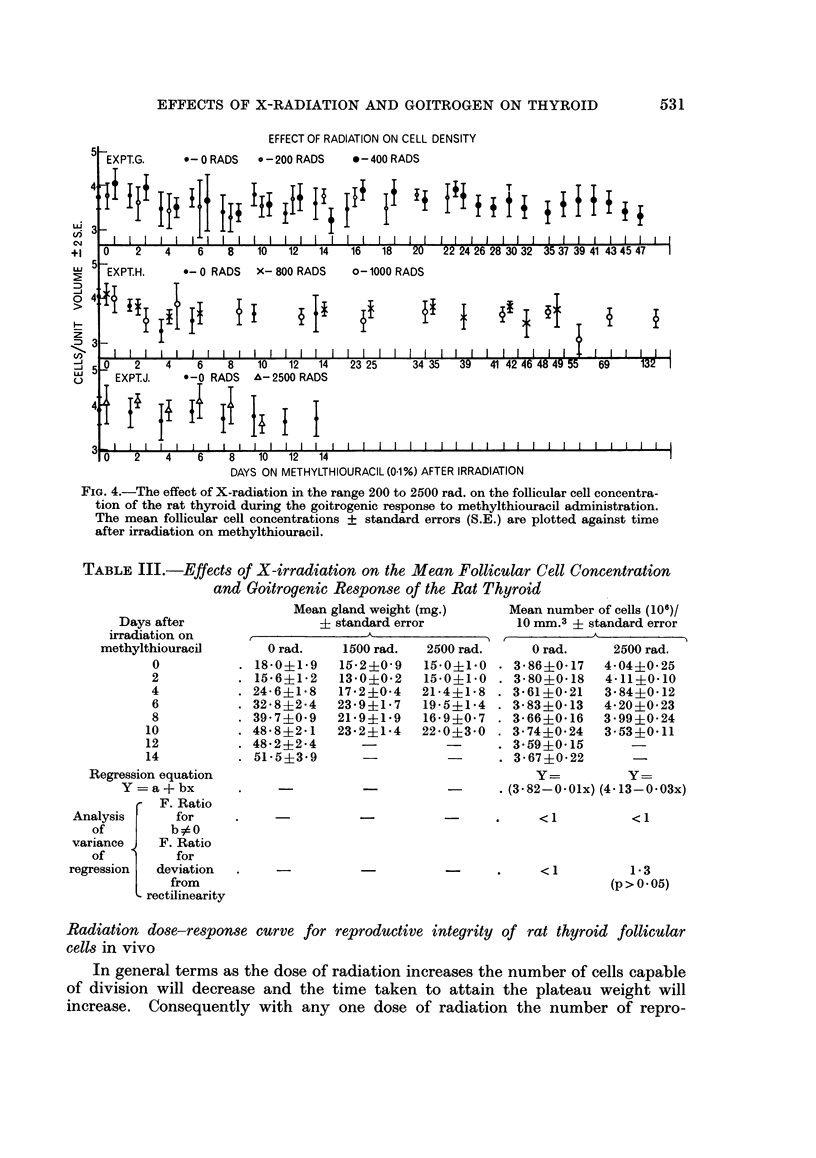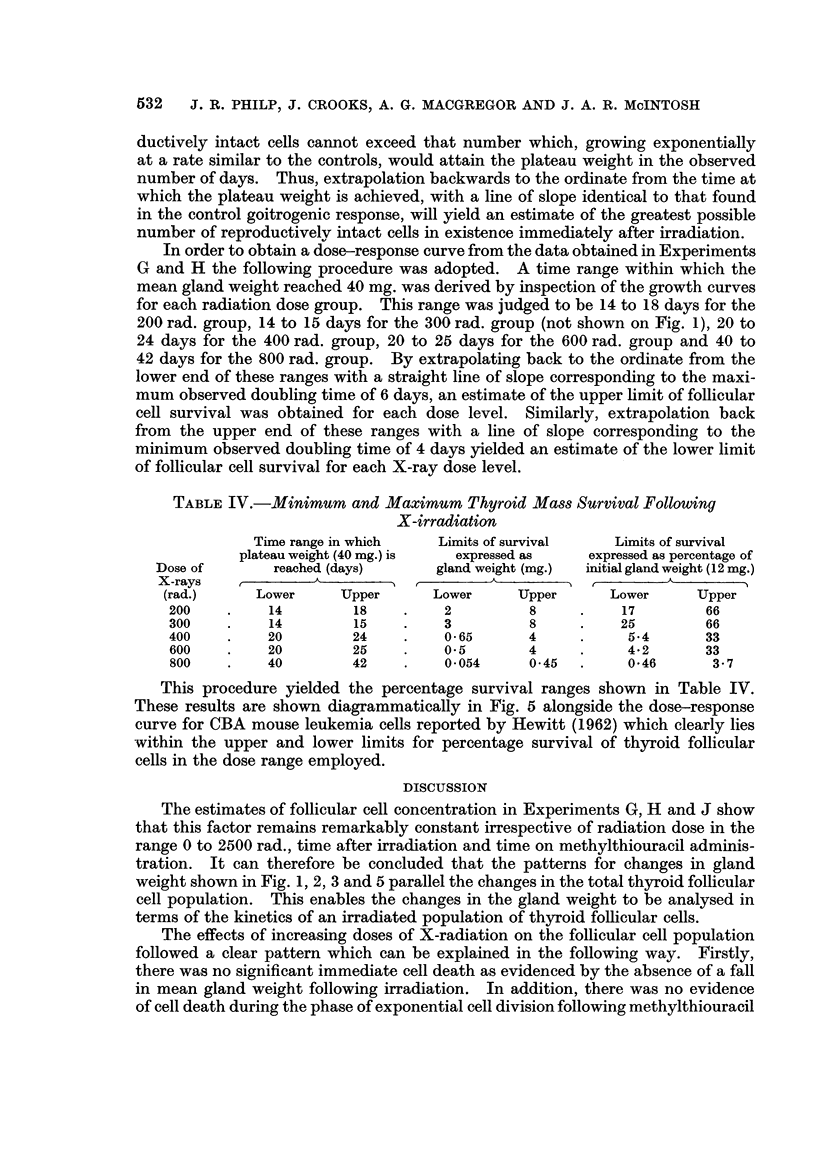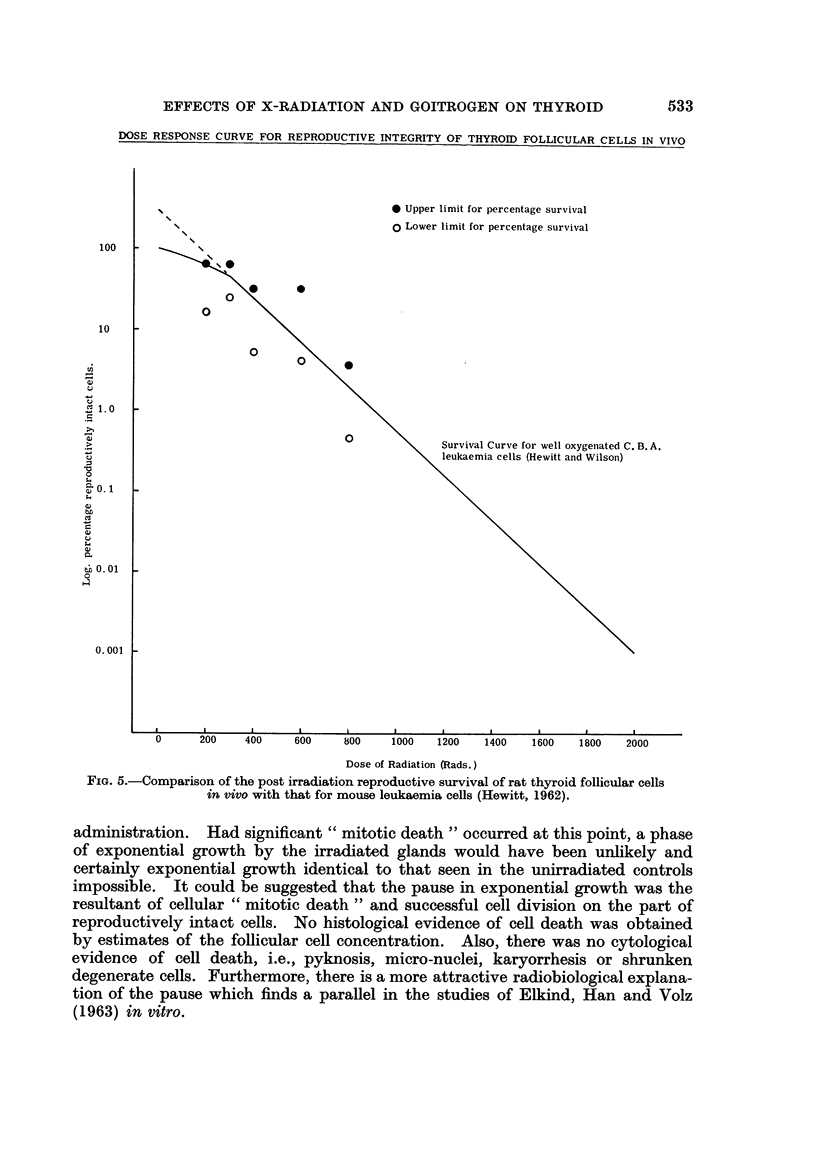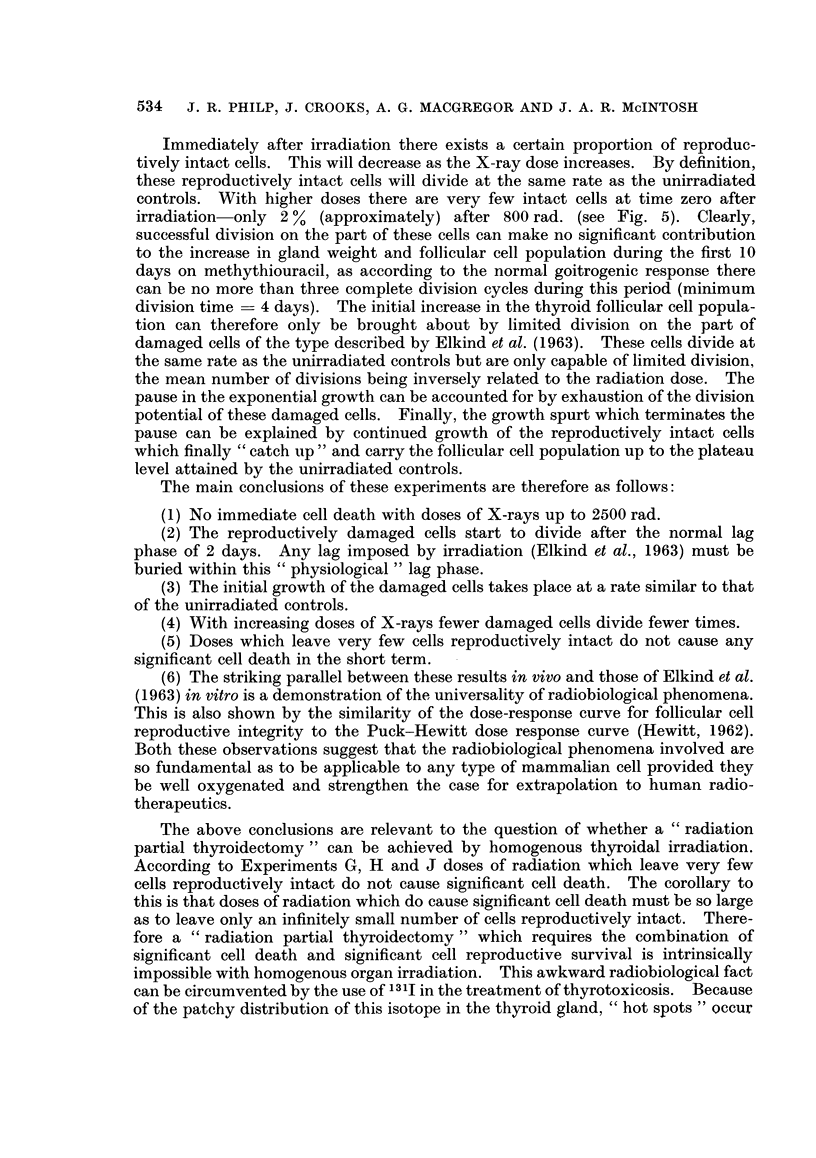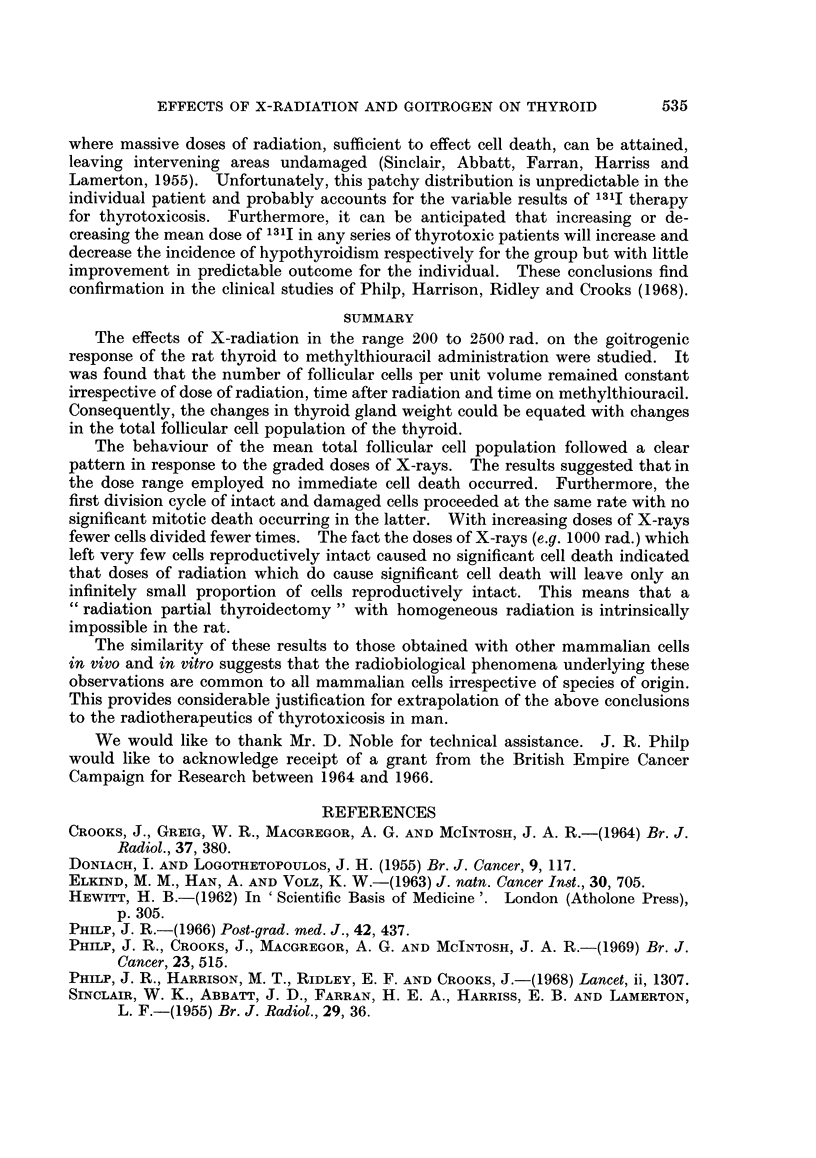# The short term effects of x-radiation on goitrogen induced growth of the rat thyroid.

**DOI:** 10.1038/bjc.1969.65

**Published:** 1969-09

**Authors:** J. R. Philp, J. Crooks, A. G. Macgregor, J. A. McIntosh


### THE SHORT TERM EFFECTS OF X-RADIATION ON GOITROGEN INDUCED GROWTH OF THE RAT THYROID

J. R. PHILP, J. CROOKS, A. G. MACGREGOR AND J. A. R. McINTOSH

*From the Department of Therapeutics and Pharmacology and the Department of Medical Physics, University Medical School, Foresterhill, Aberdeen*

Received for publication December 13, 1968

A REVIEW of the radiobiology of the thyroid gland has already been published by Philp (1966). In relation to the present study, Doniach and Logothetopoulos (1955) used graded doses of radioiodine ($^{131}I$) to study the effects of ionising radiation on the rat thyroid. They found that doses of $^{131}I$ which markedly reduced the reproductive capacity of the thyroid cells under a goitrogenic stimulus did not impair their ability to hypertrophy and the capacity of the whole gland to function normally. Crooks, Greig, Macgregor and McIntosh (1964) measured the degree of inhibition of the goitrogenic response of the rat thyroid to methylthiouracil administration brought about by various accurately measured doses of X-rays from an external source. They found progressive inhibition of the goitrogenic response with increasing doses of X-rays, although the uptake of a tracer dose of $^{131}I$ was unaffected by doses up to 1600 rad. In view of these observations, it was thought that the model described by Philp, Crooks, Macgregor and McIntosh (1969), in which the follicular cells of the rat thyroid divide exponentially as a result of methylthiouracil administration, would provide an interesting system for quantitative investigation of the effects of X-radiation on cell viability and reproductive capacity *in vivo*. In particular it was hoped to assess the possibility of a "radiation partial thyroidectomy" or its functional equivalent in the rat, because of the relevance of this question to the treatment of hyperthyroidism with $^{131}I$.

### MATERIALS AND METHODS

Doses of X-rays in the range 200 to 2500 rad. were delivered to the rat thyroid from a Westinghouse 250 kv machine with an output of 25 rad. per minute obtained with 180 kv, 12·14 mA and a 0·5 mm. copper filter. The rats were anaesthetised by an intraperitoneal injection of 1·5 to 3 ml. freshly prepared 2·5 % tribromethylalcohol solution (Avertin) according to the length of time required for the irradiation. The rats' bodies were covered by a lead shield in which holes had been cut overlying the position of the thyroid gland.

Fresh aliquots of 0·1 % methylthiouracil in 1 % sucrose were prepared every second day (Philp *et al.*, 1969). In addition, a suspension of methylthiouracil powder in 0·85 % saline was prepared to a concentration of 25 mg./ml. Immediately following irradiation each animal received a subcutaneous injection of 1 ml. of this suspension. This procedure insured a uniform starting point for thyroid stimulation as many of the animals were drowsy during the first few hours after anaesthesia and could not be relied on to drink the 0·1 % methythiouracil solution with which their drinking water had been replaced. In each radiation dose category random groups of animals were killed at intervals after irradiation on methylthiouracil, their thyroid glands removed, cleaned and weighed and estimates of the mean follicular cell concentration carried out as already described (Philp *et al.*, 1969).

*Experiment G:* Adult male Wistar rats weighing 200 g. to 280 g. were allocated at random into 68 groups of 7 each. Ten groups were used to determine the normal (control) goitrogenic growth response curve. The animals were each given a subcutaneous injection of 25 mg. methylthiouracil and their drinking water replaced by the 0·1 % methylthiouracil solution. Two groups (14 animals) were killed on day zero and one group on days 2, 4, 6, 8, 10, 12, 14 and 16 after the commencement of methylthiouracil administration.

Thirteen groups received a dose of 200 rad. to their thyroid glands and were started on methylthiouracil: 2 of these groups were killed on day zero and one group on alternate days up to 22 days after irradiation. Twenty groups received a dose of 300 rad., 2 of which were killed on day zero and one group on days 2, 4, 6, 8, 9, 11, 13, 15, 17, 19, 21, 23, 25, 27, 29, 31, 33 and 35 after irradiation on methylthiouracil. Finally, 25 groups of animals received 400 rad. to their thyroids 2 of which were killed on day zero and one group on alternate days up to 32 days and then on days 35, 37, 39, 41, 43, 45 and 47.

*Experiment H:* This experiment was similar to Experiment G except for the doses of radiation employed. The animals were once more constituted into random groups of 7 each. Ten groups (controls) received methylthiouracil only, 19 groups received 600 rad., 18 groups received 800 rad. and 24 groups received 1000 rad. to their thyroids and were started on methylthiouracil in the usual way. The days on which the animals were killed can be seen in the results (Table II).

*Experiment J:* In this experiment, 18 groups of 6 animals each were used to study the effects of doses of 1500 rad. and 2500 rad. on the goitrogenic response. Six groups were used to obtain the control (unirradiated) goitrogenic response, 6 groups received 1500 rad. and 6 groups 2500 rad. to their thyroids. Methylthiouracil was administered as usual and one group from each category killed on days 0, 2, 4, 6, 8 and 10 after irradiation. Pilot studies had shown that many rats receiving doses of 1500 to 2500 rad. died 14–20 days after irradiation. Consequently these experiments were not prolonged beyond 10 days after irradiation.

As this series of experiments proceeded it became clear that it would be sufficient to select sample groups of animals for follicular cell concentration (F.C.C.) estimations (Philp *et. al.*, 1969). Those groups on which estimates of the F.C.C. were made and indicated in the results.

### RESULTS

### *The effects of X-rays on the goitrogenic response*

*Experiment G:* In this experiment the effects of doses of 200 rad. and 400 rad. X-irradiation on the goitrogenic response of the rat thyroid to methylthiouracil administration were observed. The results are contained in Table I and shown diagrammatically in Fig. 1. In the unirradiated (control) goitrogenic response there was the usual lag phase of approximately 2 days followed by exponential growth in gland weight for 8 to 10 days until a plateau was reached (Philp *et. al.*,

1969). In the irradiated groups the pattern of a lag phase of 2 days followed by initial exponential growth identical to that in the control goitrogenic response was observed. However, as a result of X-irradiation it can be seen that the exponential growth phase is interrupted by a pause during which there is little or no increase in gland weight. The pause is temporary, however, and is terminated by a resumption of growth until the plateau weight attained by the controls is reached. It should be noted that the pause occurs earlier and lasts longer in the

FIG. 1—The effects of 200 rad. and 400 rad. X-radiation on the goitrogenic response to methylthiouracil administration. The log mean gland weights ± standard errors (S.E.) are plotted against time on methylthiouracil after irradiation.

400 rad. group compared to the 200 rad. group. As an approximation arrived at by eye the pauses in exponential growth in the 200 rad. and 400 rad. groups began at 33 mg. and 30 mg. respectively. The plateau weight was finally attained at between 14 and 18 days in the 200 rad. group and between 20 and 24 days in the 400 rad. group.

*Experiment H:* In this experiment the effects of doses of 600 rad., 800 rad. and 1000 rad. on the goitrogenic response were studied. The results are contained in Table II and shown diagrammatically in Fig. 2. In the irradiated groups, as in Experiment G, there is a lag phase of approximately 2 days followed by initial exponential growth at a rate similar to that found in the controls. Once more, the pause in exponential growth occurred earlier and lasted longer with increasing doses of X-rays. In the 1000, 800 and 600 rad. groups the pause commenced at gland weights of approximately 27 mg., 25 mg. and 22 mg. respectively. In the 600 rad. group the plateau weight was reached at between 20 and 25 days after irradiation and in the 800 rad. group at between 40 and 42 days. In the 1000 rad. group, as far as could be determined, the pause continued even up to 132 days after irradiation on methylthiouracil.

*Experiment J:* In this experiment the effects of doses of 1500 and 2500 rad. on the goitrogenic response were studied. As mentioned previously these experiments were continued for only 10 days after irradiation because many of the

FIG. 2.—The effects of 600 rad, 800 rad. and 1000 rad. X-radiation on the goitrogenic response to methylthiouracil administration. The log mean gland weights ± standard errors (S.E.) are plotted against time on methylthiouracil after irradiation.

animals treated with doses of this order died 14 to 20 days after radiation as a result of pharyngitis and pneumonia. The results are contained in Table III, and shown graphically in Fig. 3. Once more the pattern is consistent with a lag phase of approximately 2 days and initial exponential growth in gland weight similar to that of the unirradiated controls. The evidence suggests that in this case the pause occurred at an approximate mean gland weight of 17 mg. in the 2500 rad. group and 20 mg. in the 1500 rad. group. Clearly no observations on the length of the pause in growth could be obtained.

*The effects of X-irradiation on the mean thyroid follicular cell concentration (F.C.C.)*

For purposes of presentation the results of Experiments G, H and J are best considered together. The results are contained in the respective parts of Tables I, II, III and are shown diagrammatically in Fig. 4, where the numbers of follicular cells per 10 mm.[3] in each experiment are plotted against time after irradiation on methylthiouracil. In each case the regression equation ($Y = a + bx$) of mean follicular cell concentration on time after irradiation on methylthiouracil was calculated and the results shown in Tables I, II, III. Analysis of variance carried out on these regression equations revealed that, irrespective of the dose of X-radiation or time after irradiation on methylthiouracil there was no significant

FIG. 3.—The effects of 1500 rad. and 2500 rad. X-radiation on the goitrogenic response to methylthiouracil administration. The log mean gland weights ± standard errors (S.E.) are plotted against time on methylthiouracil after irradiation.

deviation from rectilinearity and that in all cases the slopes of the regression equations (b) did not differ significantly from zero. Therefore with doses of X-rays in the range 200 to 2500 rad. there was no significant change in the mean number of follicular cells per unit volume following goitrogenic challenge with methylthiouracil. A further analysis of variance was performed on all the data from Experiments G, H and J in order to determine the regression of the mean follicular cell concentration on radiation dose. This demonstrated that the regression equation $Y = 3{\cdot}72 + 0{\cdot}001x$ had no significant slope ($F < 1$) and showed no significant deviation from rectilinearity ($F = 3{\cdot}6$; $P > 0{\cdot}05$). Therefore doses of X-rays in the range of 0 to 2500 rad. did not affect the mean follicular cell concentration.

TABLE I.—*Effects of X-irradiation on the Mean Follicular Cell Concentration and Goitrogenic Response of the Rat Thyroid*

| Days after irradiation on methylthiouracil | Mean gland weight (mg.) ± standard error | | | | Mean number of cells $(10^6)/10$ mm.$^3$ ± standard error | | |
|---|---|---|---|---|---|---|---|
| | 0 rad. | 200 rad. | 300 rad. | 400 rad. | 0 rad. | 200 rad. | 400 rad. |
| 0 | 12·7±0·7 | 11·9±0·6 | 14·0±0·3 | 13·0±1·0 | 3·68±0·13 | 4·81±0·58 | 4·05±0·21 |
| 2 | 13·7±0·7 | 13·1±1·1 | 14·4±1·0 | 12·7±0·8 | 3·74±0·14 | 3·56±0·20 | 3·94±0·17 |
| 4 | 17·1±1·2 | 16·8±1·5 | 20·7±0·8 | 16·7±1·1 | 3·43±0·19 | 3·38±0·19 | 3·45±0·11 |
| 6 | 24·6±1·4 | 23·2±0·8 | 23·8±1·3 | 26·3±0·11 | 3·68±0·11 | 3·47±0·32 | 3·62±0·33 |
| 8 | 32·5±1·8 | 26·9±1·9 | 26·5±1·6 | 28·8±2·7 | 3·35±0·20 | 3·24±0·15 | 3·33±0·10 |
| 9 | — | — | 31·5±1·2 | — | — | — | — |
| 10 | 37·2±1·7 | 33·7±2·6 | — | 30·0±2·6 | 3·55±0·14 | 3·47±0·12 | 3·53±0·13 |
| 11 | — | — | 30·6±1·8 | — | — | — | — |
| 12 | 41·2±2·0 | 34·3±2·7 | — | 33·5±1·0 | 3·32±0·11 | 3·63±0·17 | 3·70±0·16 |
| 13 | — | — | 34·1±3·1 | — | — | — | — |
| 14 | 52·5±4·9 | 38·5±1·7 | — | 43·2±4·4 | 3·54±0·18 | 3·72±0·08 | 3·16±0·13 |
| 15 | — | — | 44·8±1·5 | — | — | — | — |
| 16 | 49·0±2·5 | 38·8±1·9 | — | 35·0±3·1 | 3·42±1·1 | 3·69±0·11 | 3·87±0·13 |
| 17 | — | — | 44·5±1·1 | — | — | — | — |
| 18 | — | 50·2±4·3 | — | 35·7±2·2 | — | 3·42±0·17 | 3·81±0·16 |
| 19 | — | — | 41·2±2·6 | — | — | — | — |
| 20 | — | 40·0±2·8 | — | 39·5±3·4 | — | 3·75±0·07 | 3·62±0·12 |
| 21 | — | — | 39·4±1·5 | — | — | — | — |
| 22 | — | 52·4±4·5 | — | 36·1±1·5 | — | 3·68±0·18 | 3·90±0·10 |
| 23 | — | — | 44·0±4·6 | — | — | — | — |
| 24 | — | — | — | 46·9±3·8 | — | — | 3·76±0·16 |
| 25 | — | — | 55·0±4·9 | — | — | — | — |
| 26 | — | — | — | 47·4±2·0 | — | — | 3·51±0·12 |
| 27 | — | — | 51·7±3·1 | — | — | — | — |
| 28 | — | — | — | 46·9±5·7 | — | — | 3·48±0·11 |
| 29 | — | — | 51·0±3·0 | — | — | — | — |
| 30 | — | — | — | 45·3±3·3 | — | — | 3·64±0·19 |
| 32 | — | — | — | 47·7±4·5 | — | — | 3·49±0·13 |
| 35 | — | — | — | 47·8±3·2 | — | — | 3·38±0·12 |
| 37 | — | — | — | 46·2±4·6 | — | — | 3·56±0·14 |
| 39 | — | — | — | 41·0±2·0 | — | — | 3·66±0·18 |
| 41 | — | — | — | 52·1±4·6 | — | — | 3·66±0·18 |
| 43 | — | — | — | 53·5±4·1 | — | — | 3·60±0·12 |
| 45 | — | — | — | 51·1±4·2 | — | — | 3·40±0·10 |
| 47 | — | — | — | 51·9±3·6 | — | — | 3·32±0·06 |
| Regression equation Y = (a + bx) | | | | | Y= (3·57−0·01x) | Y= (3·55+0·00x) | Y= (3·77−0·01x) |
| Analysis of variance of regression: F. Ratio for b ≠0 | | | | | <1 | <1 | <1 |
| Analysis of variance of regression: F. Ratio for deviation from rectilinearity | | | | | <1 | <1 | <1 |

TABLE II.—*Effects of X-irradiation on the Mean Follicular Cell Concentration and Goitrogenic Response of the Rat Thyroid*

| Days after irradiation on methylthiouracil | Mean gland weight (mg.) ±standard error | | | | Mean number of cells ($10^6$)/10 mm.$^3$ ±standard error | | |
|---|---|---|---|---|---|---|---|
| | 0 rad. | 600 rad. | 800 rad. | 1000 rad. | 0 rad. | 800 rad. | 1000 rad. |
| 0 | 13·1±0·6 | 12·6±0·6 | 10·4±0·5 | 10·4±0·7 | 4·42±0·10 | 4·12±0·15 | 3·09±0·17 |
| 2 | 12·3±0·8 | 12·1±0·7 | 14·1±1·3 | 12·5±1·2 | 3·82±0·15 | 3·79±0·10 | 3·47±0·13 |
| 4 | 16·9±0·5 | 14·0±1·2 | 16·5±0·7 | 12·8±1·2 | 3·25±0·03 | 3·55±0·08 | 3·85±0·28 |
| 6 | 26·2±2·0 | 27·5±2·5 | 26·2±1·2 | 19·8±1·7 | 3·50±0·15 | 3·63±0·13 | — |
| 8 | 39·0±2·2 | 30·0±1·5 | 27·2±1·2 | 25·1±2·9 | — | — | 3·06±0·12 |
| 10 | 40·8±2·3 | 34·2±2·8 | 31·0±1·6 | 22·2±1·6 | 3·65±0·09 | — | — |
| 12 | 41·4±1·9 | 41·0±2·8 | — | 28·5±2·7 | — | — | 3·39±0·18 |
| 14 | 52·3±3·1 | 34·1±2·4 | 33·3±1·9 | — | 3·77±0·21 | 3·71±0·10 | — |
| 16 | 50·5±3·4 | — | — | 31·0±1·3 | — | — | — |
| 17 | — | 40·6±2·7 | — | — | — | — | — |
| 18 | — | — | 34·7±2·1 | — | — | — | — |
| 20 | — | 43·9±4·4 | — | 28·0±2·0 | — | — | — |
| 21 | — | — | 37·7±2·8 | — | — | — | — |
| 23 | — | — | — | 29·3±1·3 | — | — | 3·44±0·11 |
| 25 | — | 41·1±2·2 | 32·6±2·8 | — | — | 3·70±0·10 | — |
| 27 | — | — | — | 29·6±3·4 | — | — | — |
| 28 | — | 42·9±3·0 | 34·4±3·4 | — | — | — | — |
| 30 | — | — | — | 32·1±1·7 | — | — | — |
| 32 | — | 38·8±4·1 | 33·9±1·5 | — | — | — | — |
| 34 | — | — | — | 26·5±1·7 | — | — | 3·64±0·13 |
| 35 | — | 46·1±4·3 | 34·6±3·3 | — | — | 3·77±0·10 | — |
| 37 | — | — | — | 31·4±2·6 | — | — | — |
| 39 | — | 45·4±3·3 | 33·6±4·1 | — | — | 3·55±0·16 | — |
| 41 | — | — | — | 26·6±1·4 | — | — | 3·59±0·09 |
| 42 | — | — | 41·1±2·2 | — | — | 3·78±0·60 | — |
| 44 | — | — | — | 30·9±2·3 | — | — | 3·65±0·07 |
| 46 | — | — | 39·6±4·0 | — | — | 3·40±0·15 | — |
| 48 | — | — | — | 29·6±1·5 | — | — | — |
| 49 | — | — | 43·4±3·2 | — | — | 3·71±0·20 | — |
| 51 | — | — | — | 34·3±3·5 | — | — | — |
| 55 | — | — | — | 22·7±0·6 | — | — | 4·06±0·15 |
| 62 | — | — | — | 24·2±0·8 | — | — | — |
| 69 | — | — | — | 34·8±2·1 | — | — | 3·52±0·10 |
| 92 | — | — | — | 25·1±2·6 | — | — | — |
| 132 | — | — | — | 36·8±9·4 | — | — | 3·50±0·10 |
| Regression equation Y = (a + bx) | | | | | Y = (4·05−0·04x) | Y = (3·87−0·01x) | Y = (3·94−0·00x) |
| Analysis of variance of regression: F. Ratio for b≠0 | | | | | 2·0 ($P>0·05$) | 1·2 ($P>0·05$) | <1 |
| Analysis of variance of regression: F. Ratio for deviation from rectilinearity | | | | | 6·1 ($P<0·01$) | <1 | <1 |

FIG. 4.—The effect of X-radiation in the range 200 to 2500 rad. on the follicular cell concentration of the rat thyroid during the goitrogenic response to methylthiouracil administration. The mean follicular cell concentrations ± standard errors (S.E.) are plotted against time after irradiation on methylthiouracil.

TABLE III.—*Effects of X-irradiation on the Mean Follicular Cell Concentration and Goitrogenic Response of the Rat Thyroid*

| Days after irradiation on methylthiouracil | Mean gland weight (mg.) ± standard error | | | Mean number of cells ($10^6$)/ 10 mm.$^3$ ± standard error | |
|---|---|---|---|---|---|
| | 0 rad. | 1500 rad. | 2500 rad. | 0 rad. | 2500 rad. |
| 0 | 18·0±1·9 | 15·2±0·9 | 15·0±1·0 | 3·86±0·17 | 4·04±0·25 |
| 2 | 15·6±1·2 | 13·0±0·2 | 15·0±1·0 | 3·80±0·18 | 4·11±0·10 |
| 4 | 24·6±1·8 | 17·2±0·4 | 21·4±1·8 | 3·61±0·21 | 3·84±0·12 |
| 6 | 32·8±2·4 | 23·9±1·7 | 19·5±1·4 | 3·83±0·13 | 4·20±0·23 |
| 8 | 39·7±0·9 | 21·9±1·9 | 16·9±0·7 | 3·66±0·16 | 3·99±0·24 |
| 10 | 48·8±2·1 | 23·2±1·4 | 22·0±3·0 | 3·74±0·24 | 3·53±0·11 |
| 12 | 48·2±2·4 | — | — | 3·59±0·15 | — |
| 14 | 51·5±3·9 | — | — | 3·67±0·22 | — |
| Regression equation Y = a + bx | — | — | — | Y= (3·82−0·01x) | Y= (4·13−0·03x) |
| Analysis of variance of regression: F. Ratio for b≠0 | — | — | — | <1 | <1 |
| Analysis of variance of regression: F. Ratio for deviation from rectilinearity | — | — | — | <1 | 1·3 (p>0·05) |

*Radiation dose–response curve for reproductive integrity of rat thyroid follicular cells* in vivo

In general terms as the dose of radiation increases the number of cells capable of division will decrease and the time taken to attain the plateau weight will increase. Consequently with any one dose of radiation the number of reproductively intact cells cannot exceed that number which, growing exponentially at a rate similar to the controls, would attain the plateau weight in the observed number of days. Thus, extrapolation backwards to the ordinate from the time at which the plateau weight is achieved, with a line of slope identical to that found in the control goitrogenic response, will yield an estimate of the greatest possible number of reproductively intact cells in existence immediately after irradiation.

In order to obtain a dose–response curve from the data obtained in Experiments G and H the following procedure was adopted. A time range within which the mean gland weight reached 40 mg. was derived by inspection of the growth curves for each radiation dose group. This range was judged to be 14 to 18 days for the 200 rad. group, 14 to 15 days for the 300 rad. group (not shown on Fig. 1), 20 to 24 days for the 400 rad. group, 20 to 25 days for the 600 rad. group and 40 to 42 days for the 800 rad. group. By extrapolating back to the ordinate from the lower end of these ranges with a straight line of slope corresponding to the maximum observed doubling time of 6 days, an estimate of the upper limit of follicular cell survival was obtained for each dose level. Similarly, extrapolation back from the upper end of these ranges with a line of slope corresponding to the minimum observed doubling time of 4 days yielded an estimate of the lower limit of follicular cell survival for each X-ray dose level.

TABLE IV.—*Minimum and Maximum Thyroid Mass Survival Following X-irradiation*

| Dose of X-rays (rad.) | Time range in which plateau weight (40 mg.) is reached (days) | | Limits of survival expressed as gland weight (mg.) | | Limits of survival expressed as percentage of initial gland weight (12 mg.) | |
|---|---|---|---|---|---|---|
| | Lower | Upper | Lower | Upper | Lower | Upper |
| 200 | 14 | 18 | 2 | 8 | 17 | 66 |
| 300 | 14 | 15 | 3 | 8 | 25 | 66 |
| 400 | 20 | 24 | 0·65 | 4 | 5·4 | 33 |
| 600 | 20 | 25 | 0·5 | 4 | 4·2 | 33 |
| 800 | 40 | 42 | 0·054 | 0·45 | 0·46 | 3·7 |

This procedure yielded the percentage survival ranges shown in Table IV. These results are shown diagrammatically in Fig. 5 alongside the dose–response curve for CBA mouse leukemia cells reported by Hewitt (1962) which clearly lies within the upper and lower limits for percentage survival of thyroid follicular cells in the dose range employed.

### DISCUSSION

The estimates of follicular cell concentration in Experiments G, H and J show that this factor remains remarkably constant irrespective of radiation dose in the range 0 to 2500 rad., time after irradiation and time on methylthiouracil administration. It can therefore be concluded that the patterns for changes in gland weight shown in Fig. 1, 2, 3 and 5 parallel the changes in the total thyroid follicular cell population. This enables the changes in the gland weight to be analysed in terms of the kinetics of an irradiated population of thyroid follicular cells.

The effects of increasing doses of X-radiation on the follicular cell population followed a clear pattern which can be explained in the following way. Firstly, there was no significant immediate cell death as evidenced by the absence of a fall in mean gland weight following irradiation. In addition, there was no evidence of cell death during the phase of exponential cell division following methylthiouracil

DOSE RESPONSE CURVE FOR REPRODUCTIVE INTEGRITY OF THYROID FOLLICULAR CELLS IN VIVO

FIG. 5.—Comparison of the post irradiation reproductive survival of rat thyroid follicular cells *in vivo* with that for mouse leukaemia cells (Hewitt, 1962).

administration. Had significant " mitotic death " occurred at this point, a phase of exponential growth by the irradiated glands would have been unlikely and certainly exponential growth identical to that seen in the unirradiated controls impossible. It could be suggested that the pause in exponential growth was the resultant of cellular " mitotic death " and successful cell division on the part of reproductively intact cells. No histological evidence of cell death was obtained by estimates of the follicular cell concentration. Also, there was no cytological evidence of cell death, i.e., pyknosis, micro-nuclei, karyorrhesis or shrunken degenerate cells. Furthermore, there is a more attractive radiobiological explanation of the pause which finds a parallel in the studies of Elkind, Han and Volz (1963) *in vitro*.

Immediately after irradiation there exists a certain proportion of reproductively intact cells. This will decrease as the X-ray dose increases. By definition, these reproductively intact cells will divide at the same rate as the unirradiated controls. With higher doses there are very few intact cells at time zero after irradiation—only 2 % (approximately) after 800 rad. (see Fig. 5). Clearly, successful division on the part of these cells can make no significant contribution to the increase in gland weight and follicular cell population during the first 10 days on methythiouracil, as according to the normal goitrogenic response there can be no more than three complete division cycles during this period (minimum division time = 4 days). The initial increase in the thyroid follicular cell population can therefore only be brought about by limited division on the part of damaged cells of the type described by Elkind *et al.* (1963). These cells divide at the same rate as the unirradiated controls but are only capable of limited division, the mean number of divisions being inversely related to the radiation dose. The pause in the exponential growth can be accounted for by exhaustion of the division potential of these damaged cells. Finally, the growth spurt which terminates the pause can be explained by continued growth of the reproductively intact cells which finally "catch up" and carry the follicular cell population up to the plateau level attained by the unirradiated controls.

The main conclusions of these experiments are therefore as follows:

(1) No immediate cell death with doses of X-rays up to 2500 rad.

(2) The reproductively damaged cells start to divide after the normal lag phase of 2 days. Any lag imposed by irradiation (Elkind *et al.*, 1963) must be buried within this " physiological " lag phase.

(3) The initial growth of the damaged cells takes place at a rate similar to that of the unirradiated controls.

(4) With increasing doses of X-rays fewer damaged cells divide fewer times.

(5) Doses which leave very few cells reproductively intact do not cause any significant cell death in the short term.

(6) The striking parallel between these results *in vivo* and those of Elkind *et al.* (1963) *in vitro* is a demonstration of the universality of radiobiological phenomena. This is also shown by the similarity of the dose-response curve for follicular cell reproductive integrity to the Puck–Hewitt dose response curve (Hewitt, 1962). Both these observations suggest that the radiobiological phenomena involved are so fundamental as to be applicable to any type of mammalian cell provided they be well oxygenated and strengthen the case for extrapolation to human radiotherapeutics.

The above conclusions are relevant to the question of whether a " radiation partial thyroidectomy " can be achieved by homogenous thyroidal irradiation. According to Experiments G, H and J doses of radiation which leave very few cells reproductively intact do not cause significant cell death. The corollary to this is that doses of radiation which do cause significant cell death must be so large as to leave only an infinitely small number of cells reproductively intact. Therefore a " radiation partial thyroidectomy " which requires the combination of significant cell death and significant cell reproductive survival is intrinsically impossible with homogenous organ irradiation. This awkward radiobiological fact can be circumvented by the use of $^{131}I$ in the treatment of thyrotoxicosis. Because of the patchy distribution of this isotope in the thyroid gland, " hot spots " occur where massive doses of radiation, sufficient to effect cell death, can be attained, leaving intervening areas undamaged (Sinclair, Abbatt, Farran, Harriss and Lamerton, 1955). Unfortunately, this patchy distribution is unpredictable in the individual patient and probably accounts for the variable results of $^{131}I$ therapy for thyrotoxicosis. Furthermore, it can be anticipated that increasing or decreasing the mean dose of $^{131}I$ in any series of thyrotoxic patients will increase and decrease the incidence of hypothyroidism respectively for the group but with little improvement in predictable outcome for the individual. These conclusions find confirmation in the clinical studies of Philp, Harrison, Ridley and Crooks (1968).

SUMMARY

The effects of X-radiation in the range 200 to 2500 rad. on the goitrogenic response of the rat thyroid to methylthiouracil administration were studied. It was found that the number of follicular cells per unit volume remained constant irrespective of dose of radiation, time after radiation and time on methylthiouracil. Consequently, the changes in thyroid gland weight could be equated with changes in the total follicular cell population of the thyroid.

The behaviour of the mean total follicular cell population followed a clear pattern in response to the graded doses of X-rays. The results suggested that in the dose range employed no immediate cell death occurred. Furthermore, the first division cycle of intact and damaged cells proceeded at the same rate with no significant mitotic death occurring in the latter. With increasing doses of X-rays fewer cells divided fewer times. The fact the doses of X-rays (*e.g.* 1000 rad.) which left very few cells reproductively intact caused no significant cell death indicated that doses of radiation which do cause significant cell death will leave only an infinitely small proportion of cells reproductively intact. This means that a " radiation partial thyroidectomy " with homogeneous radiation is intrinsically impossible in the rat.

The similarity of these results to those obtained with other mammalian cells *in vivo* and *in vitro* suggests that the radiobiological phenomena underlying these observations are common to all mammalian cells irrespective of species of origin. This provides considerable justification for extrapolation of the above conclusions to the radiotherapeutics of thyrotoxicosis in man.

We would like to thank Mr. D. Noble for technical assistance. J. R. Philp would like to acknowledge receipt of a grant from the British Empire Cancer Campaign for Research between 1964 and 1966.

### REFERENCES

CROOKS, J., GREIG, W. R., MACGREGOR, A. G. AND MCINTOSH, J. A. R.—(1964) *Br. J. Radiol.*, **37**, 380.

DONIACH, I. AND LOGOTHETOPOULOS, J. H. (1955) *Br. J. Cancer*, **9**, 117.

ELKIND, M. M., HAN, A. AND VOLZ, K. W.—(1963) *J. natn. Cancer Inst.*, **30**, 705.

HEWITT, H. B.—(1962) In ' Scientific Basis of Medicine '. London (Atholone Press), p. 305.

PHILP, J. R.—(1966) *Post-grad. med. J.*, **42**, 437.

PHILP, J. R., CROOKS, J., MACGREGOR, A. G. AND MCINTOSH, J. A. R.—(1969) *Br. J. Cancer*, **23**, 515.

PHILP, J. R., HARRISON, M. T., RIDLEY, E. F. AND CROOKS, J.—(1968) *Lancet*, ii, 1307.

SINCLAIR, W. K., ABBATT, J. D., FARRAN, H. E. A., HARRISS, E. B. AND LAMERTON, L. F.—(1955) *Br. J. Radiol.*, **29**, 36.